# Platelet-rich plasma for the treatment of discogenic low back pain: a prospective randomized controlled trial

**DOI:** 10.3389/fpain.2025.1648772

**Published:** 2025-08-13

**Authors:** Xiangyi Wang, Siqi Wang, Jie Zhang, Gang Xie, Jin Zhang

**Affiliations:** ^1^ Department of Rehabilitation and Pain, Zigong Fourth People’s Hospital, Zigong, Sichuan, China; ^2^ Department of Radiology, Zigong Fourth People’s Hospital, Zigong, Sichuan, China

**Keywords:** discogenic low back pain, platelet-rich plasma, Pfirrmann grading system, magnetic resonance imaging, apparent diffusion coefficient, Japanese Orthopaedic Association, visual analog scale

## Abstract

**Objective:**

This study aimed to compare the therapeutic effect of PRP and methylene blue injection in patients with discogenic low back pain.

**Methods:**

A total of 40 patients with discogenic low back pain were randomly divided into two groups, with 20 patients in group A receiving platelet-rich plasma injections and 20 patients in group B receiving methylene blue injections. Visual analog scale (VAS) scores, Japanese Orthopaedic Association (JOA) scores, Pfirrmann grades, and MRI apparent diffusion coefficients (ADCs) were recorded in both groups before the injections and 6 months after the injections.

**Results:**

Compared with group B, the postoperative VAS score of group A was significantly decreased, while the JOA score and ADC score were significantly increased (*P* < 0.05). There was no significant difference in Pfirrmann grade between the two groups after surgery (*P* > 0.05). In group A, the Pfirrmann grade after surgery was lower than before surgery (*P* < 0.05), and the ADC score was higher than before surgery (*P* < 0.05). There was no significant difference in Pfirrmann grade for the patients in group B before and after surgery (*P* > 0.05), and their ADC score was lower than that before surgery (*P* < 0.05).

**Conclusion:**

Compared with a methylene blue injection, platelet-rich plasma can significantly reduce pain, improve the function of the lumbar spine, increase the diffusion ability of water molecules in the intervertebral disc, and improve the degree of intervertebral discogenic degeneration in patients with discogenic low back pain.

## 1 Introduction

Chronic low back pain ranks first in global disease burden, with discogenic low back pain accounting for approximately 40% and tending to occur more frequently in younger patients (1). Discogenic low back pain refers to intractable low back pain caused by lumbar discogenic degeneration. Currently, most of the treatment methods are minimally invasive interventional therapies, such as radiofrequency thermocoagulation, ozone injection, and methylene blue injection. According to its pathogenesis, these treatments can eliminate its symptoms, but do not improve the underlying degenerative changes. In recent years, *in vitro* and *in vivo* studies and clinical data have proven the safety and effectiveness of platelet-rich plasma (PRP) in the treatment of discogenic low back pain, inhibiting disc degeneration and delaying the disease process (2). However, higher-quality evidence and studies on discogenic morphological changes are lacking. In addition, the authors' previous studies found that patients with discogenic low back pain with complete rupture of the annulus on discography may also experience epidural leakage during an injection of methylene blue, and the drugs remaining in the disc do not play a therapeutic role (3). Therefore, 40 patients with discogenic low back pain who did not leak contrast agent into the spinal canal during an epidural were selected and randomly (https://www.random.org) divided into two groups. Group A received platelet-rich plasma injections and group B received methylene blue injections. Thus, this study aimed to explore the therapeutic effect of these two methods and observe the morphological changes of the intervertebral disc under MRI before and after the injections, so as to provide a clinical reference for the treatment of discogenic low back pain.

## 2 Methods

### 2.1 Research objects

**Figure 1:**
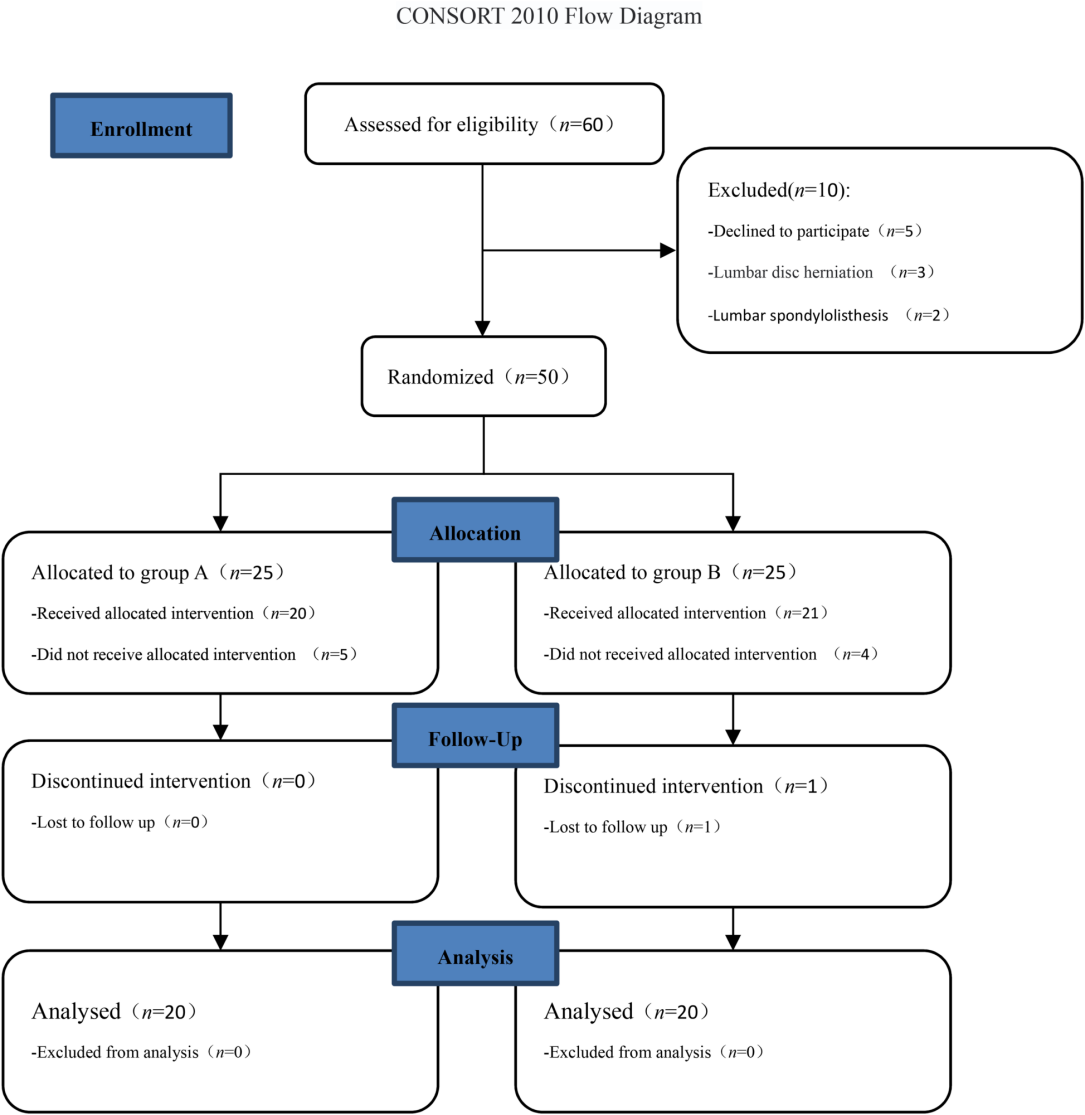
CONSORT 2010 flow diagram.

This study was a prospective, randomized controlled study and was reviewed by the Ethics Committee of Zigong Fourth People's Hospital (ethics approval number: EC-20220172-01) and registered with the China Clinical Trial Center (registration number: ChiCTR2400085027). Patients with discogenic low back pain who received injection treatments at Zigong Fourth People's Hospital from June 2024 to September 2024 were selected for the study. All patients provided signed informed consent. This study adhered to the CONSORT guidelines (Figure 1).

#### 2.1.1 Inclusion criteria

The inclusion criteria were as follows: patients with clinical diagnosis of discogenic low back pain; low signal in the intervertebral disc with high signal at the posterior margin of the intervertebral disc on lumbar MRI T2 lipomatography; age between 18 and 65 years; total course of disease of more than 6 months, conservative treatment was ineffective, and a visual analog scale (VAS) score of ≥4; imaging examination showed no obvious lumbar spondylolisthesis, lumbar disc herniation, lumbar spinal stenosis, lumbar fracture, and lumbar tumor.

#### 2.1.2 Exclusion criteria

The exclusion criteria were as follows: the patient or their family requested a withdrawal; puncture failure; patients with an annulus rupture on discography and leakage of contrast media in the spinal canal epidural; patients with mental illness who could not cooperate; patients with communication disorders; patients who had infected puncture sites; patients with cardiopulmonary diseases who could not tolerate surgery; and patients with abnormal coagulation function.

### 2.2 Preoperative preparation

The operation was performed under local anesthesia. A fluoroscopic operating table and a prone frame were used during the operation. Group A was injected with platelet-rich plasma: 10 mL of venous blood was taken from the body, centrifuged at 4,500 RPM for 10 min, and 2 mL of platelet-rich plasma was prepared with sodium citrate as the anticoagulant. Group B was injected with 1 mL/10 mg of methylene blue.

### 2.3 Surgical methods

The patient was placed in a prone position and positioned under C-arm fluoroscopy before surgery. The median posterior spinal line and the horizontal line of the targeted intervertebral space were marked. The puncture point was located on the horizontal line and was opened 6–8 cm away from the symptomatic side. The insertion angle was parallel to the upper and lower endplates of the targeted intervertebral body. A puncture between the fifth lumbar vertebra (L5) and the first sacral vertebra (S1) avoids any obstruction by the iliac crest and allows for an appropriate puncture angle with the upper and lower endplates. With the assistance of C-arm fluoroscopy and local anesthesia, an 18-gauge puncture needle was inserted into the intervertebral disc through the Kambin safety triangle. The tip of the needle was placed near the center of the intervertebral disc on the C-arm fluoroscopy film, and at the back 1/3 of the intervertebral disc on the lateral film. No blood or cerebrospinal fluid was extracted from the syringe. Then, 1 mL of iohexol was injected at a rate of 0.5 mL/ s to perform a discography. If the intervertebral disc in question induces a reproduction of the original pain, and at least one adjacent disc does not induce a reproduction of the original pain, the discography is positive (4). At this time, the morphology of the disc was observed, and the patients with incomplete annulus fibrosa rupture and no contrast agent leakage in the spinal canal epidural were randomly divided into groups A and B (https://www.random.org). After the completion of the injections, the puncture needles were removed, and after observation for adverse reactions, the patients’ wounds were disinfected with iodophor and covered with a sterile dressing.

**Figure 2:**
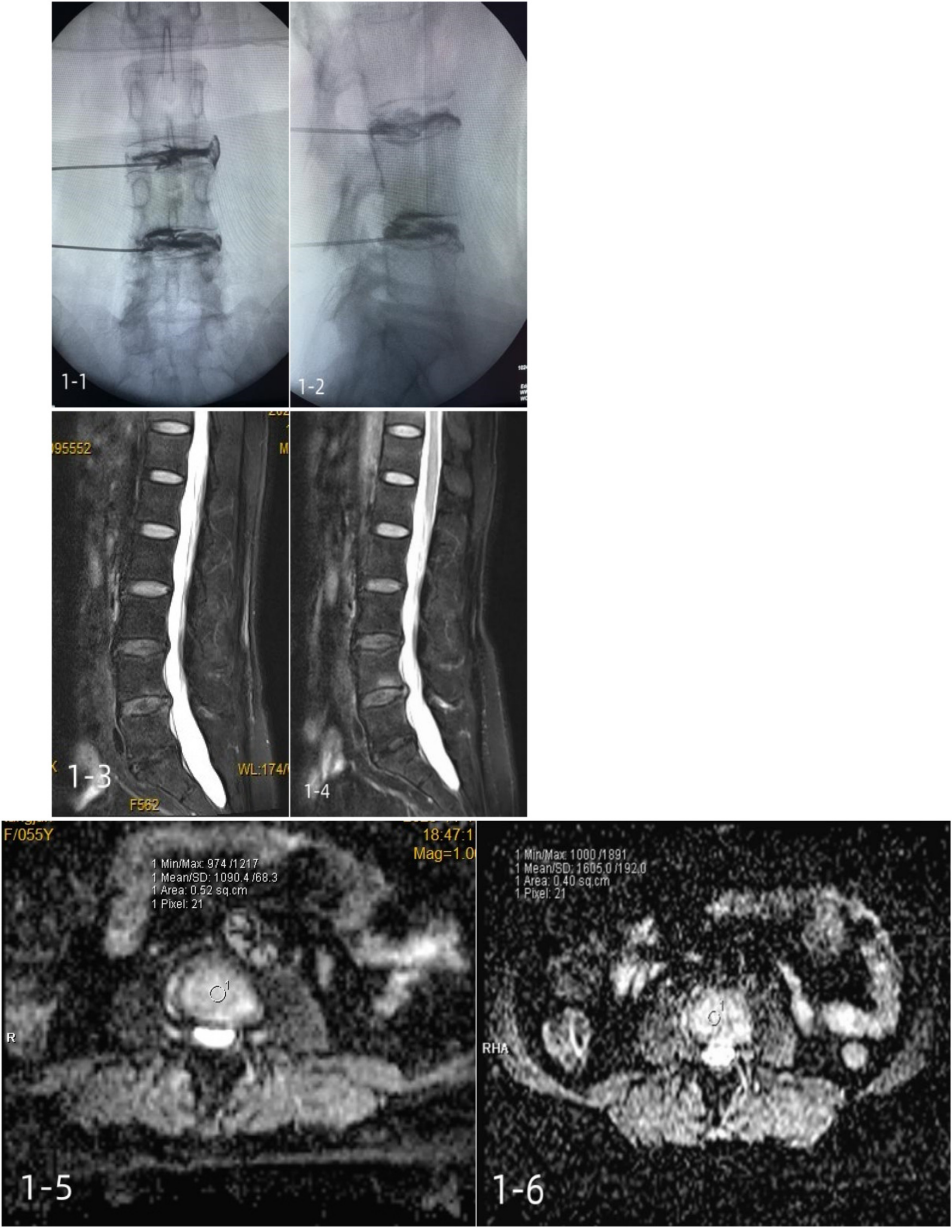
A 55-year-old female patient with a positive lumbar discography and incomplete annulus fibrosus rupture was treated with a platelet-rich plasma injection. 1-1, 1-2: intraoperative contrast anterior-lateral film; 1-3: preoperative Pfirrmann grade III; 1-4: postoperative Pfirrmann grade III; 1-5: Preoperative mean ADC of 1,090.4 ± 68.3; 1-6: mean postoperative ADC of 1,605.0 ± 192.0.

In group A, 1 mL of platelet-rich plasma was injected using a puncture needle at a uniform rate. A typical case is shown in Figure 2.

**Figure 3:**
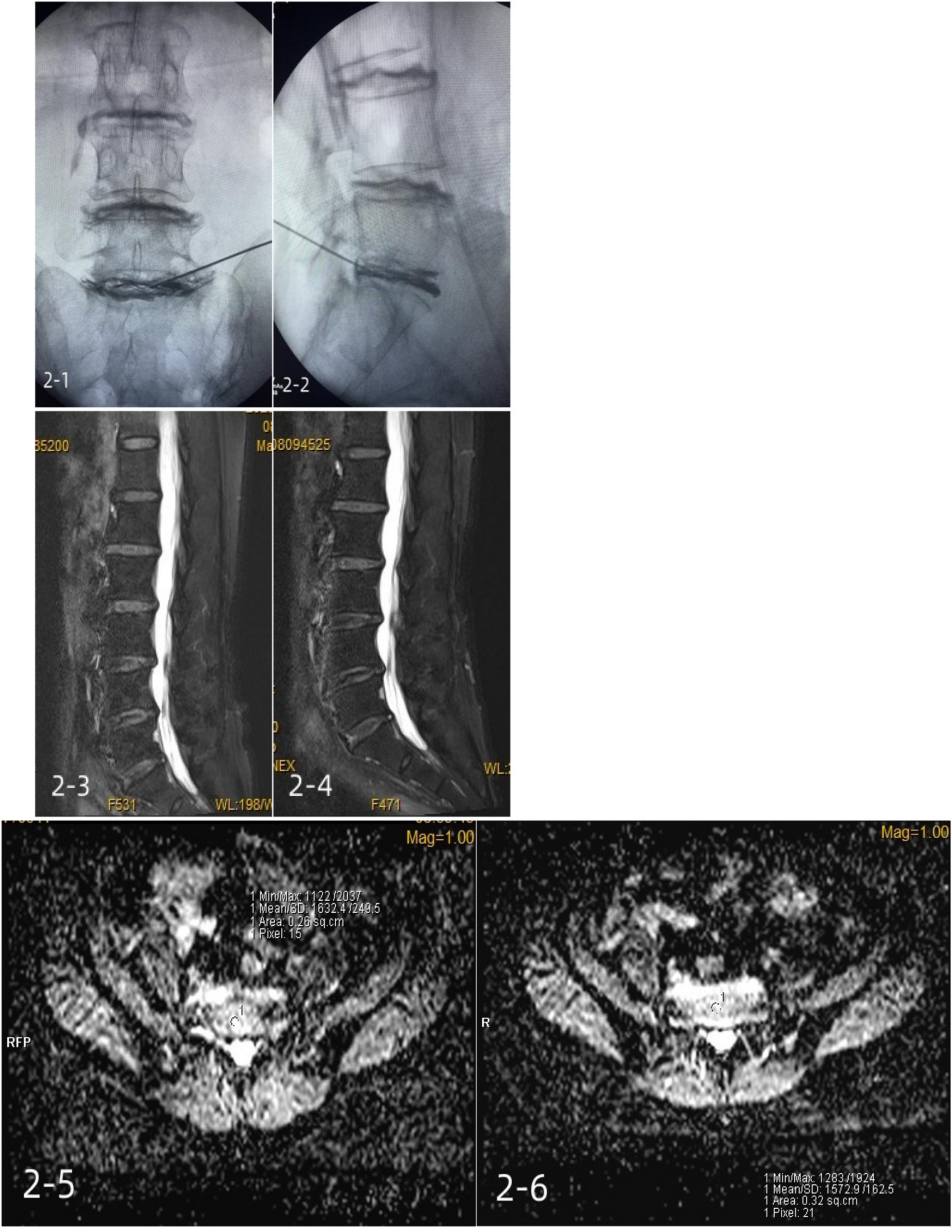
A 64-year-old female patient with positive lumbar (L5/S1) discography and incomplete annulus fibrosus rupture was treated with a methylene blue injection. 2-1, 2-2: intraoperative contrast anterior-lateral film; 2-3: preoperative Pfirrmann grade III; 2-4: postoperative Pfirrmann grade III; 2-5: preoperative mean ADC of 1,632.4 ± 249.5; 2-6: mean postoperative ADC of 1,572.9 ± 162.5.

In group B, 1 mL/10 mg of methylene blue was injected using a puncture needle at a uniform rate. A typical case is shown in Figure 3.

### 2.4 Postoperative procedure

The patients were encouraged to be mobile, while wearing a waist belt, after 4 h in bed. Core muscle strength exercises, such as semi-squat against the wall, plank support, and supine knee lift, were performed from the first day after surgery. Waist belts were worn for 2 weeks after the surgery. The patients were recommended to avoid strenuous exercise and prolonged standing for 3 months after surgery, and MRI was conducted and reviewed 6 months after surgery.

### 2.5 Evaluation indices

#### 2.5.1 Visual analog scale

The VAS is scored from 0 to 10 points, with 0 denoting no pain and 10 denoting the most pain.

#### 2.5.2 Japanese Orthopaedic Association scoring system

The highest score in the Japanese Orthopaedic Association (JOA) scoring system is 29 and the lowest is 0. The lower the score, the more obvious the dysfunction. The JOA treatment improvement rate (as a percentage) was calculated as follows: (postoperative score − preoperative score)/(29 − preoperative score)×100. Thus, 0%–24% indicated poor improvement, 25%–49% indicated medium improvement, 50%–74% indicated good improvement, and more than 75% indicated excellent improvement.

#### 2.5.3 Pfirrmann grades on lumbar MRI sagittal T2 lipomatosis (5)

The Pfirrman grading system is as follows: in Grade Ⅰ, the intervertebral disc structure shows uniform white high signal, the boundary between the nucleus pulposus and the annulus fibrosus is obvious, and the disc height is normal; in Grade II, the intervertebral disc structure presents an uneven white high signal with or without gray horizontal bands, the boundary between the nucleus pulposus and the annulus fibrosus is obvious, and the disc height is normal; in Grade III, the intervertebral disc structure shows uneven gray signal intensity, the boundary between the nucleus pulposus and the annulus fibrosus is not obvious, and there is a slight decrease in disc height; in Grade IV, the disc structure shows uneven gray or black low signal, the boundary between the nucleus pulposus and annulus fibrosus has disappeared, and the intervertebral disc height has decreased moderately; and in Grade V, there is uneven black low signal in the intervertebral disc structure, the boundary between the nucleus pulposus and the annulus fibrosus has disappeared, and the vertebral space has collapsed.

#### 2.5.4 Magnetic resonance imaging apparent diffusion coefficient

The system transmits the MRI diffusion-weighted imaging (DWI) data of the intervertebral disc to the syngo.via workstation to automatically calculate the apparent diffusion coefficient (ADC) map. The ADC values of the nucleus pulposus of each intervertebral disc were measured on the ADC images at the transverse axis center level. During the measurement, the nucleus pulposus region in the central part of the intervertebral disc was selected. The region of interest size was 25–55 mm^2^ and the shape was circular. The measurement was repeated three times for each intervertebral disc and the average value was calculated.

### 2.6 Statistical analysis

SPSS 23.0 software was used for the statistical analysis. Measurement data were expressed as ($\bar{x}\pm s$). The paired sample *t*-test was used for intra-group comparisons and the independent sample *t*-test was used for inter-group comparisons. The *x*^2^ test was used for counting data comparisons. *P* < 0.05 was considered statistically significant.

## 3 Results

In total, 40 patients (Figure 1) were included in the final statistical analysis, including 18 men and 22 women, aged 26–64 years. They were randomly divided into two groups according to the order of admission: (1) the platelet-rich plasma injection group (group A) had 20 cases, including 11 men and 9 women, aged 28–64 (43.8 ± 11.3) years; and (2) the methylene blue injection group (group B) had 20 patients, including 7 men and 13 women, aged 26–63 (45.5 ± 11.2). There were no significant differences in age and gender between the two groups (*P* > 0.05).

The preoperative VAS scores, JOA scores, Pfirrmann grades, and ADC scores were not significantly different between the two groups (*P* > 0.05).

**Figure 4:**
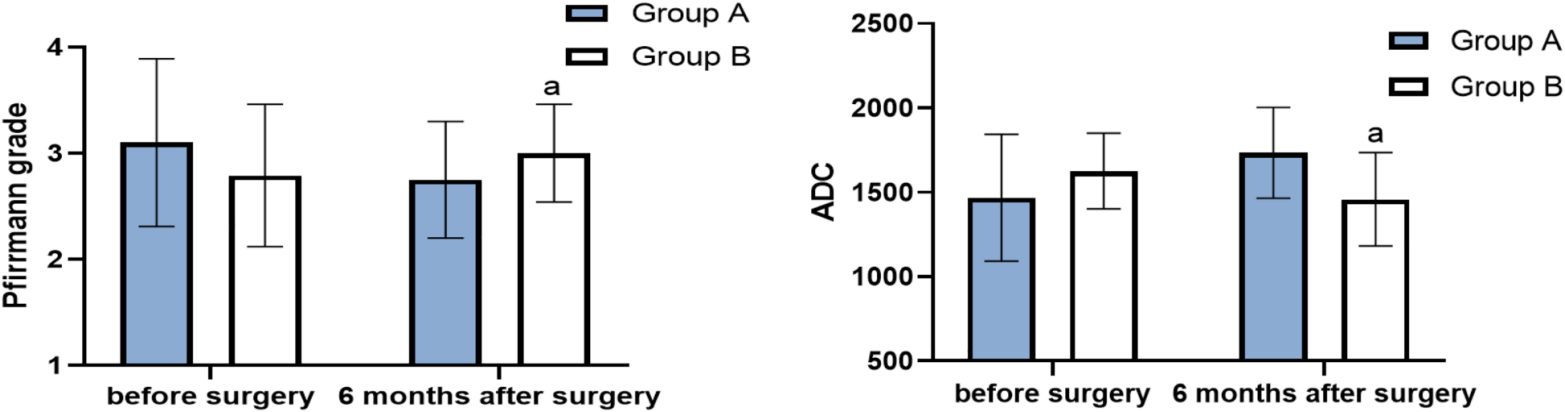
Pfirrmann grade and ADC before surgery and 6 months after surgery (score, $\bar{x}\pm s$) (compared with group A, ^a^*P* < 0.05).

There was no significant difference in Pfirrmann grade between the two groups (*P* > 0.05). In group A, the Pfirrmann grade at 6 months after surgery was lower than that before surgery (*P* < 0.05), and the ADC score at 6 months after surgery was higher than that before surgery (*P* < 0.05). There was no significant difference in Pfirrmann grade for the patients in group B before and after surgery (*P* > 0.05), and their ADC score at 6 months after surgery was lower than that before surgery (*P* < 0.05) (Table 1, Figure 4). No significant vascular and nerve injury or infection complications occurred in the two groups.

**Table 1:** Pfirrmann grade and ADC before surgery and 6 months after surgery (score, $\bar{x}\pm s$).

Groups	Pfirrmann grade		ADC	
	Before surgery	6 months after surgery	Before surgery	6 months after surgery
Group A (*n* = 20)	3.10 ± 0.79	2.75 ± 0.55	1,468.0 ± 375.4	1,734.0 ± 269.0
Group B (*n* = 20)	2.79 ± 0.67	3.00 ± 0.46	1,625.7 ± 224.2	1,458.5 ± 276.3
t-value	1.06	1.56	1.61	3.20
*P*-value	0.33	0.13	0.12	0.00

**Figure 5:**
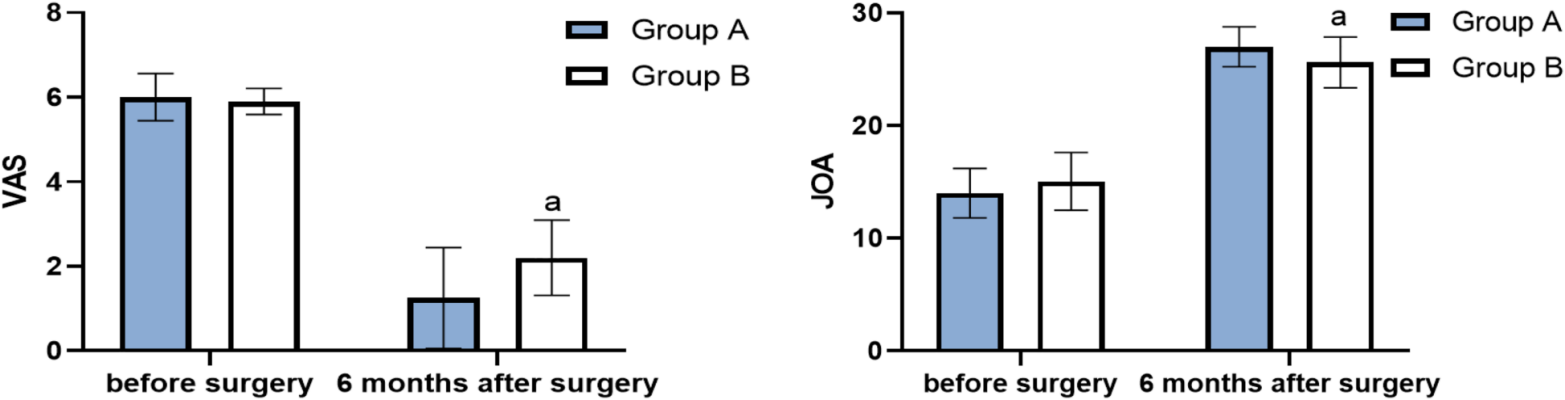
VAS and JOA before surgery and 6 months after surgery (score, $\bar{x}\pm s$) (compared with group A, ^a^*P* < 0.05).

The VAS score of group A at 6 months after surgery was significantly lower than that of group B (*P* < 0.05), and the JOA score of group A at 3 months after surgery was significantly higher than that of group B (*P* < 0.05) (Table 2, Figure 5). The average JOA score improvement rate in group A was 86.7% ± 10.9%, and the excellent rate was 100%. The average of group B was 75.0% ± 17.3%, and the excellent and good rate was 95%. The difference between the two groups was statistically significant (t = 2.5398, *P* < 0.05).

**Table 2:** VAS and JOA scores before surgery and 6 months after surgery (score, $\bar{x}\pm s$).

Groups	VAS		JOA	
	Before surgery	6 months after surgery	Before surgery	6 months after surgery
Group A (*n* = 20)	6.00 ± 0.56	1.25 ± 1.19	14.00 ± 2.20	27.00 ± 1.78
Group B (*n* = 20)	5.90 ± 0.31	2.20 ± 0.89	15.05 ± 2.56	25.60 ± 2.26
t-value	0.70	2.97	1.39	2.18
*P*-value	0.49	0.01	0.17	0.03

## 4 Discussion

There are five typical clinical features of discogenic low back pain: decreased sitting tolerance, limited stretching ability, difficulty in holding weight, inability to maintain a fixed position for a long time, and increased lower back pain after movement. The cause of the pain could be discogenic degeneration, the compression of nerve endings in the new granulation tissue after an annulus fibrosus tear, and/or nociceptive receptors receiving and transmitting pain stimulation signals. Furthermore, pressure changes in the intervertebral disc, the production of inflammatory mediators, and stimulation of abnormal peripheral nerves cause pain. An intervertebral disc is a type of vascularized tissue, which completes the renewal of nutrients and substances through passive diffusion, and is slow to repair itself after degeneration or injury. In the past, the treatment of discogenic low back pain was mainly conducted through a local puncture in the intervertebral disc using chemical methods, such as an injection of methylene blue, or physical methods, such as radiofrequency thermocoagulation, which have been used to destroy abnormal growing nerve endings and block pain conduction to reduce symptoms (6). A previous study (7) found that contrast media can also cause disc degeneration, and the higher the dose, the higher the degree of disc degeneration. However, the North American Spinal Surgery Association states that discography is the only way to diagnose discogenic low back pain (8). Although there is some controversy, it can reproduce the pain and intuitively show the degree of disc degeneration, and thus plays an irreplaceable role in diagnosis. Iodohexol imaging has the least toxic effect on nucleus pulposus cells, and the injection rate should be controlled to 0.5 mL/second, the injection volume should be 1 mL, and the total amount should not exceed 3.5 mL (9).

At present, MRI is a commonly used auxiliary means for diagnosing discogenic low back pain. Pfirrmann grading based on MRI image analysis is the most commonly used grading method for evaluating disc degeneration and provides a simple and relatively reliable evaluation standard for clinical work. However, there are certain subjective biases, especially when evaluating the degree of disc degeneration in the elderly. It is difficult to distinguish between grades III and IV (5). MRI DWI is currently the only MRI technology that reflects the diffusion movement of water molecules. The ADC is commonly used to reflect the microscopic movement of water molecules in tissues. The smaller the ADC value, the lower the water content in the nucleus pulposus of the intervertebral disc, the more difficult the diffusion of water molecules, and the higher the degree of intervertebral disc degeneration. The ADC value is significantly negatively correlated with Pfirrmann grading of intervertebral discs, and the combination of the two can provide a more accurate and quantitative evaluation of intervertebral discs (10).

PRP is autologous blood with a higher platelet concentration than the physiological baseline and is obtained through centrifugation. In recent years, PRP has been widely used in the regeneration and repair of tissues such as tendons, ligaments, and cartilage. With the deepening of research, its application in intervertebral disc degeneration has gradually increased. Given the pathogenesis of discogenic low back pain, reducing the stimulation of inflammation on nerve endings, enhancing the regenerative ability of the intervertebral disc, repairing the intervertebral disc, increasing the water content, and improving the ability of the intervertebral disc to resist pressure are key to obtaining an ideal curative effect. Platelet-rich plasma contains a large number of growth factors that can promote stem cell differentiation, histiocytic proliferation, matrix synthesis, and a variety of anti-inflammatory factors, which can reduce inflammation. Furthermore, chemokines in PRP can regulate the immune response and promote tissue healing and regeneration (11). Compared to other regenerative medicine applications, such as growth factors and stem cells, PRP is simple to manufacture and use, is low-cost, and can be isolated from autologous sources without an immune reaction.

In this study, no nerve injury, inflammation, or infection occurred after the injections of platelet-rich plasma, which further indicates that a platelet-rich plasma injection is a safe method. The postoperative VAS and JOA scores of group A were significantly improved compared with those before the surgery, which confirmed the effectiveness of platelet-rich plasma. In this study, a randomized controlled clinical study was used to compare PRP injection with methylene blue injection (the control group), a commonly used injection treatment. There was no significant difference in the VAS and JOA scores between the two groups before surgery. At 6 months after surgery, the VAS score of group A was significantly lower than that of group B, and the JOA score of group A was significantly higher than that of group B, with statistical significance. Moreover, the difference in the JOA treatment improvement rate between the two groups was statistically significant. Compared with methylene blue, platelet-rich plasma can significantly reduce pain and improve lumbar function.

The Pfirrmann grade in group A at 6 months after surgery was significantly higher than that before surgery, and there was no statistical difference in group B before and after surgery. The ADC value of the intervertebral disc in group A at 6 months after surgery was higher than before surgery, while that in group B at 6 months after surgery was lower than before surgery, indicating that platelet-rich plasma plays a role in increasing water content of the nucleus pulposus, repairing the intervertebral disc, and reversing degeneration in terms of intervertebral disc morphology. The degeneration of the intervertebral disc was accelerated in the methylene blue group due to the dual factors of methylene blue and the contrast media.

## 5 Conclusion

In conclusion, platelet-rich plasma is more effective than methylene blue in patients with discogenic low back pain with incomplete discogenic annulus rupture, as it increases the diffusion ability of water molecules in the intervertebral disc and improves the degree of intervertebral disc degeneration. However, the number of cases in this study was small, the follow-up time was short, patients with intervertebral disc annulus rupture were not included in the study, and there was no uniform standard for the preparation of the platelet-rich plasma or for the number, frequency, and volume of the injections, so further in-depth research is needed.

## Data Availability

The original contributions presented in the study are included in the article/Supplementary Material, further inquiries can be directed to the corresponding author.

## References

[1] Wu A March L Zheng X Huang J Wang X Zhao J Global low back pain prevalence and years lived with disability from 1990 to 2017: estimates from the Global Burden of Disease Study 2017. Ann Transl Med. (2020) 8(6):299. 10.21037/atm.2020.02.175 32355743 PMC7186678

[2] Yongan W Bo L Qiang Z Wang J Li X. Current status and prospects of platelet-rich plasma in the treatment of lumbar intervertebral disc degeneration. Rheumatol Arthritis. (2022) 11(6):77–80. 10.3969/j.issn.2095-4174.2022.06.020

[3] Xiangyi W Shiwei L Jin Z Chao W. Comparison of the efficacy of radiofrequency thermocoagulation and methylene blue injection for disc-derived low back pain. J Pract Orthop. (2022) 28(4):313–6. 10.13795/j.cnki.sgkz.2022.04.020

[4] Xiangyi W Yuejuan L Shiwei L Liu R. Transforaminal contrast-enhanced radiculography guided dural hormone injection for lumbar disc herniation. J Clin Orthop. (2020) 23(1):31–6. 10.3969/j.issn.1008-0287.2020.01.009

[5] Pfirrmann CW Metzdorf A Zanetti M Hodler J Boos N. Magnetic resonance classification of lumbar intervertebral disc degeneration. Spine (Phila Pa 1976). (2001) 26(17):1873–8. 10.1097/00007632-200109010-00011 11568697

[6] Min Z Fei'e Z. Progress of autologous platelet-rich plasma for the treatment of disc-derived low back pain. Chin J Pain Med. (2024) 30(2):131–6. 10.3969/j.issn.1006-9852.2024.02.009

[7] Weiheng W Jie ST Xiaodong H Meng Q Ye X. Effect of iodioxanol dose in disgraphy on disc degeneration in rats. J Spinal Surg. (2020) 18(5):325–30. 10.3969/j.issn.1672-2957.2020.05.008

[8] Boswell MV Trescot AM Datta S Schultz DM Hansen HC Abdi S Interventional techniques: evidence-based practice guidelines in the management of chronic spinal pain. Pain Phys. (2007) 10(1):7–111. 17256025

[9] Xilin L Xiaojian Y. Progress in lumbar spine discography. Int J Bone Sci. (2015) 36(4):273–6. 10.3969/j.issn.1673-7083.2015.04.008

[10] Yu HJ Bahri S Gardner V Muftuler LT. *In vivo* quantification of lumbar disc degeneration: assessment of ADC value using a degenerative scoring system based on Pfirrmann framework. Eur Spine J. (2015) 24(11):2442–8. 10.1007/s00586-014-3721-0 25502000

[11] Yang S Xiaofei L Shengnan M Jin C Wang L Xie W. Progress in platelet-rich plasma for intervertebral disc degeneration. Chin J Spinal Cord. (2020) 30(08):762–6. 10.3969/j.issn.1004-406X.2020.08.15

